# Using machine learning techniques for rationalising phenotypic readouts from a rat sleeping model

**DOI:** 10.1186/1758-2946-5-S1-P34

**Published:** 2013-03-22

**Authors:** Georgios Drakakis, Alexios Koutsoukas, Suzanne Clare Brewerton, David DE Evans, Andreas Bender

**Affiliations:** 1 Unilever Centre for Molecular informatics, Department of Chemistry, University of Cambridge, Lensfield Road, Cambridge, CB1 2EW, UK; 2 Eli Lilly and Company Limited, Erl Wood Manor, Sunninghill Road, Windlesham, Surrey, GU20 6PH, UK

## 

Understanding the mode of action of small molecules is critical for drug research, both with respect to efficacy and anticipated side effects. Given that many compounds act on multiple targets simultaneously, it appears that linking single targets to outcomes is no longer sufficient. Hence, in this work we explore machine learning methods for rationalising phenotypic readouts from a rat model for hypnotics based on a polypharmacology approach. We hypothesise that by combining target prediction and machine learning techniques we are able to derive information regarding the mode of action of small molecules. In particular, we applied this hypothesis on a subset of the Eli Lilly SCORE™ dataset. This comprised 845 data instances, each consisting of 7 phenotypic readouts which attribute towards a good sleeping pattern. We employed a target prediction tool[1] to anticipate bioactivities of ligands in combination with the CN2 and C4.5 machine learning algorithms to derive interpretable rule lists and classification trees for the observed phenotypes. A review of the known mechanisms of action for the largest categories of hypnotics suggests that our results are in most cases consistent with current literature on the mode of action of hypnotics. This suggests that our method can potentially yield significant information regarding the mode of action of hypnotics, and in addition novel targets that are not yet well-established in literature. As further applications of this work, we are currently preparing to apply our methodology to a subset of a Traditional Chinese Medicine dataset and a phenotypic screening dataset for Xenopus.
